# Mapping sites of high TB transmission risk: Integrating the shared air and social behaviour of TB cases and adolescents in a South African township

**DOI:** 10.1016/j.scitotenv.2017.01.026

**Published:** 2017-04-01

**Authors:** Benjamin Patterson, Carl D Morrow, Daniel Kohls, Caroline Deignan, Samuel Ginsburg, Robin Wood

**Affiliations:** a Division of Infectious Diseases, Columbia University, College of Physicians and Surgeons, New York, NY, USA; b Institute of Infectious Disease and Molecular Medicine (IDM), Faculty of Health Sciences, University of Cape Town, Cape Town, South Africa; c Desmond Tutu HIV Centre, IDM, University of Cape Town, Cape Town, South Africa; d Department of Electrical Engineering, Faculty of Engineering & the Built Environment, University of Cape Town, South Africa

**Keywords:** Tuberculosis, Adolescents, GIS, Transmission, South Africa, Hot spot

## Abstract

**Background:**

Tuberculosis remains a major public health problem in poverty-stricken areas of the world. Communal gathering places account for the majority of TB transmission in high burden settings.

**Objective:**

To investigate the social behaviour patterns of individuals who have developed TB disease and adolescents at risk of infection. To develop a cheap and effective method to locate transmission hot spots in high burden communities.

**Design:**

Portable, combined CO_2_/GIS monitors and location diaries were given to individuals from a South African township. The three groups: newly diagnosed TB patients, recently treated TB patients and adolescents recorded their activities over a median of two days. Rebreathed air volumes (RAVs) at all GIS locations were calculated from CO_2_ levels using the Rudnick-Milton variant of the Wells-Riley TB transmission model. Hot spot analysis was performed to determine the communal buildings which correspond to spatially clustered high RAVs.

**Results:**

Analysis of diaries found that the adolescent group spent greater time in congregate settings compared with the other two groups driven by time spent in school/work (new TB: 1%, recent TB: 8%, and adolescents: 23%). Adolescents also changed their location more frequently (9.0, 6.0, 14.3 changes per day; *p* < 0.001). The RAVs reflected this divergence between the groups (44, 40, 127 l; *p* < 0.001). Communal buildings associated with high RAVs were found to be a clinic, two schools and a library. Hot spot analysis revealed the most intense clustering of high RAVs at a community school.

**Conclusion:**

Our study demonstrates a new methodology to uncover TB transmission hot spots using a technique that avoids the need to pre-select locations. Investigation of a South African township highlighted the high risk potential of schools and high risk social behaviour of adolescents. Consequently the targeting of transmission reduction strategies to schools may prove highly efficacious in high burden settings.

## Highlights

• A novel technique to locate congregate sites of TB transmission is presented.
• CO_2_/GIS monitors were given to TB cases and adolescents from a high burden community.
• The social behaviour of the adolescent group increased their risk of exposure.
• The community school was found to be a potential TB transmission hot spot.

## Graphical abstract

Getis-Ord-Gi* cluster analysis highlighting the school as a TB transmission hot spot.

**Figure:**
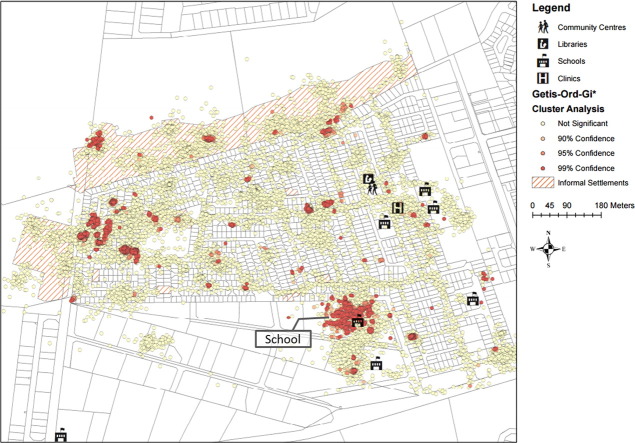

## 1 Introduction

Global TB prevalence is declining at a rate of 1.5% per annum (WHO, 2014). Nevertheless there are still an estimated 9.6 million new TB case notifications each year (WHO, 2014). In South Africa the rates of TB infection are similar to those of 100 years ago driven by a force of infection estimated to be 4–8% (Wood et al., 2011). This is a rate greater than the pre-chemotherapy era in the industrialised world (Hermans et al., 2015). The WHO strategy of active case finding is resource intensive and has not been shown to have a significant effect on TB epidemiology (Kranzer et al., 2013). In order to have such an impact interventions aimed at reducing the effective contact rate are required (Yates et al., 2016).

Airborne spread of TB demands sufficient physical proximity to share a breathing zone between an infectious case and a susceptible individual. Necessarily this locates transmission events in indoor poorly ventilated buildings frequented by infectious individuals. For adults molecular epidemiology studies (Verver et al., 2004, Glynn et al., 2015, Brooks-Pollock et al., 2011, Buu et al., 2010) together with modelling of social interaction and environmental data (Andrews et al., 2014) point to congregate settings as the most frequent location of such events in highly burdened regions. Household transmission remains significant in childhood (Middelkoop et al., 2009) but becomes less important with age and the positive association between TB infection and residential exposure to adult TB cases is lost by adolescence (> 15 years) (Middelkoop et al., 2014).

The estimation of building ventilation can be achieved by the use of CO_2_ monitoring provided the presence of a CO_2_ source (Menzies et al., 1995). Conveniently humans are highly reliable CO_2_ sources with tight regulation of concentration (4%) and an approximate adult production rate of 6 l/min. Linkage to site specific information for room occupancy allows calculation of the rebreathed air fraction for any individual within that room (Wood et al., 2014). From this fraction the Rudnick-Milton variant (Rudnick and Milton, 2003) on the Wells-Riley model gives an approximately linear relationship between rebreathed air fraction and probability of a new TB infection in a susceptible individual assuming the presence of one or more infectors. The efficacy of this approach has been demonstrated in public health field work. In an open-plan office in London incident TB infections following a three month exposure to an infectious staff member were predicted with reasonable accuracy by measuring daily CO_2_ levels and assuming an infectious quanta production rate of 13 particles/h (Pankhurst et al., 2012).

For any given community the high risk sites may vary considerably. A low cost, effective technique to identify communal buildings for targeted transmission reduction interventions would be highly desirable. Our study investigated a community with frequent transmission events as demonstrated by an undiagnosed prevalence rate > 2000 per 100,000 (Wood et al., 2007) and a latent TB infection rate that reaches 88.0% by age 31–35 (Wood et al., 2010). We sought to characterise the social behaviour of members of this community: both those who had progressed to TB disease and those who are at risk of doing so. In addition we used personal monitoring devices to map CO_2_ levels combined with diary reported room occupant numbers to derive average rebreathed air volumes (RAVs). By linking these data to GIS locations we constructed a geospatial map which could be used to identify sites of TB transmission risk.

## 2 Methods

### 2.1 Study population

All participants were resident in Masiphumelele a small, peri-urban township 40 km south of Cape Town with an estimated population of 18,000 and a TB notification rate of 2000 per 100,000 (Middelkoop et al., 2015). The study took place during the winter months (May–October) with data collected from 2012 to 2015. Pulmonary TB patients (*n* = 12) were recruited at the time of diagnosis from a clinic in Masiphumelele township. Another group of recently treated pulmonary TB patients (*n* = 24) were recruited from the same clinic shortly after finishing treatment. The median time between completion of treatment and enrolment in the study was 45.5 days (IQR 31–65). The final group were healthy adolescents from the community (*n* = 58) recruited from the Desmond Tutu Youth Centre in Masiphumelele. Enrolment in the study included both the diary section and the CO_2_/GIS monitor section.

### 2.2 Data collection

Each participant completed a questionnaire recording demographic and household details. A continuous diary recording locations visited and number of people present was kept. This was maintained for several consecutive days per participant (median time period of 2 days).

No diary information was recorded for sleep by five members of the adolescent group who recorded time overnight as spent in their own household consequently they were excluded from analysis of sleep but data on their mobility was retained.

### 2.3 GIS/CO_2_ monitoring

For the GIS/CO_2_ monitoring, participants were requested to carry a small personal device (previously described (Wood et al., 2014)) at all time throughout the study. The monitor logged values every minute for both GPS coordinates and CO_2_ in parts per million (PPM). These data were later uploaded from the device for analysis.

**Fig. 1:**
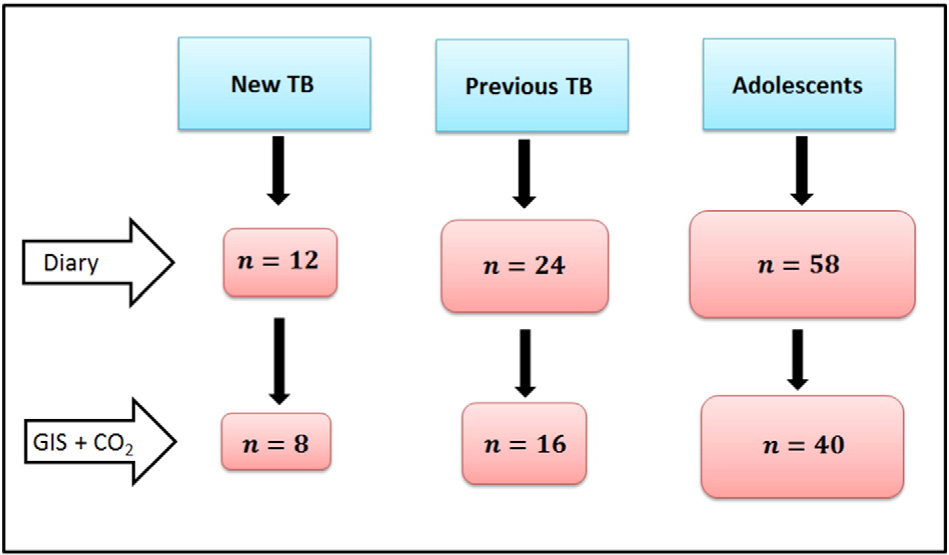
Consort diagram showing the numbers of participants for analysed for the different components of the study in the three groups.

A minimum threshold of 1100 data points in 24 h was set representing location monitoring for > 75% of each day (maximum possible 1440 min/day). Not all the monitors logged sufficient data to be included in the analysis and so data from these devices were discarded (Fig. 1). Failure to log data was attributed to either loss of signal, firmware failure or device battery failure with a resultant loss of approximately one third of participants.

To preserve battery life the GIS monitor was fitted with a motion detector such that when stationary for > 20 min it turned off. Once movement restarted the monitor turned back on and data recording quickly resumed. This typically occurred during sleeping hours but not during the rest of the day. In order to maintain proportionate location data for mapping purposes and RAV analysis the missing data points were extrapolated from the last known GPS coordinates.

On preliminary testing the GIS monitor was found to have a dither such that successive coordinates for the location of a stationary device varied by a distance of between 0.08 and 10.4 m with a mean of 2.1 m.

### 2.4 Quantification of rebreathed air volume

The Wells-Riley equation uses a Poisson process to model TB infection risk and a modified version allows for fraction of rebreathed air to approximate room ventilation. Conveniently this can be derived from environmental CO_2_ measurement. Location and time specific CO_2_ data were analysed in conjunction with diary entries for the number of people present and contributing to the CO_2_ concentration. Excess CO_2_ levels can thereby determine the local fraction of rebreathed air (described previously (Wood et al., 2014)).

### 2.5 Data analysis

To assess difference between the three groups univariate analyses were applied to the demographic characteristics, employment status, housing type, number of cohabitees, number of room co-occupants for sleeping, daily movements, sleep time and RAVs. The Fisher exact test was used for categorical variables and continuous variables were compared with *t*-tests for the normally distributed variables and Wilcoxon rank sum tests when not normally distributed.

Statistical analysis was performed using R Core Team (2015) (R: A language and environment for statistical computing. R Foundation for Statistical Computing, Vienna, Austria. URL https://www.R-project.org/). GIS mapping and hot spot analysis was performed using ESRI 2011. ArcGIS Desktop: Release 10. Redlands, CA: Environmental Systems Research Institute.

### 2.6 GIS analysis

ArcGIS (Ver.10.4, ESRI Inc., CA, USA) was used to map the GIS coordinates for the different groups investigated, these locations were linked to calculated rebreathed air volumes. Initial analysis was based on setting a threshold of RAV to select only the locations linked to higher values. All GIS linked data points were then subjected to a hot spot analysis (Environmental Systems Research Institute, 2016) to assess for local aggregation of high RAVs performed by a spatial analysis tool embedded in ArcGIS software. Euclidean distance between neighbouring points was used to set the band parameter to 5 m. The Getis-Ords Gi* statistic (Getis and Ord, 1992) was then applied to identify clusters of values that are higher or lower than expected by chance giving the output as a z score. From this a confidence level is established for the significance of high RAV clustering and given a colour grade. Statistically significant clustering was defined as Gi* *p* value < 0.05 and Gi* z score > 1.96. Cold spots were excluded from resulting images generated since these are largely outdoor congregate locations and therefore not relevant for transmission risk.

### 2.7 Ethics statement

Ethical approval was obtained from the University of Cape Town Faculty of Health Sciences Human Research Ethics Committee. All adults participating in the study provided written informed consent and all participants younger than 18 years old assented and had written consent provided by a parent or guardian. Locations of individual houses were not accurately identifiable due to the GPS dither.

## 3 Results

Demographic questionnaires, diaries and monitors were analysed and differences between the three groups were compared (Table 1). Data extracted from the questionnaire revealed that 73.4% of study participants lived in an informal housing type with no statistically significant difference between the groups. Differences were significant with respect to the mean number of cohabitees for the new TB, previous TB and adolescent groups (1.5, 2.8, 3.7 respectively; *p* = 0.004) and mean number of those sharing a room to sleep (0.4, 1.2, 1.4 respectively; *p* = 0.024).

### 3.1 Diaries

**Fig. 2:**
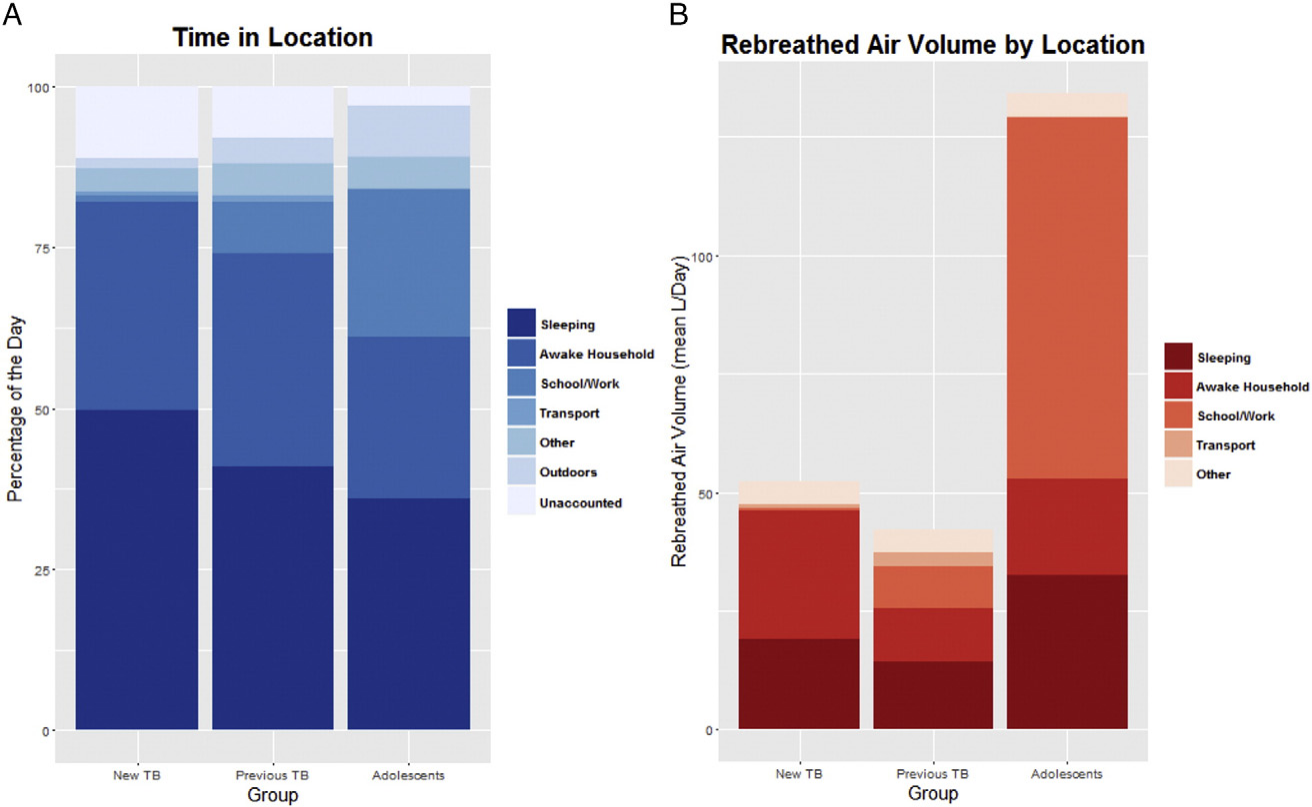
A. Comparison of the three groups in terms of proportion of the day spent at different locations. B. Comparison of the three groups in terms of mean RAVs in litres per day.

All Indoor locations documented were amalgamated into five categories: sleeping location, households, school or work, transport and all other locations combined. Time spent outside was included in a separate category and the unaccounted time in the diaries between the start and the end of the time investigated was calculated (Fig. 2A).

The new TB group reported sleeping longer compared with both the previous TB and adolescent groups (12.0, 9.9, and 9.5 h respectively; *p* < 0.001). Movement analysis was carried out by determining the daily location changes for each participant based on the number of different locations recorded. The movements per day were found to be greater in the new TB group than previous TB but far less than adolescents (8.8, 5.3 and 12.8 respectively; *p* < 0.001). The new TB group spent much less time at work or school compared with the other two (mean 1%, 8%, 23% respectively). Similarly the new TB group reported only 16.7% full time work compared with 37.5% for the previous TB group whereas 96% of the adolescents attended school.

### 3.2 Rebreathed air volume

RAVs were calculated for each individual at each location visited and incorporated into the same five categories. Total daily RAVs were similar in the new and previous TB groups but markedly elevated in the adolescent group (median 44, 40, 127 l respectively; *p* < 0.001) which was mainly accounted for by time spent outside of the household (median 0.0, 3.1, 71.3 l respectively; *p* < 0.001) (Fig. 2B).

### 3.3 External CO_2_ sources

The use of an open flame for either cooking or heating was monitored in the diaries. In household locations flame use was reported for heating by 17.5% of study participants, and for cooking by 22.0%. In all locations outside the household flame use was reported for heating by 6.4% of the participants and for cooking by 10.6%. Table 2 shows the differences between the groups. There was no statistically significant correlation found between flame use and mean CO_2_ level for household (H) or congregate (C) locations for either heating (H) or cooking (C) (HH: *p* = 0.64; HC: *p* = 0.68; CH = 0.97; CC = 0.66).

### 3.4 Mapping of hot spots

**Fig. 3:**
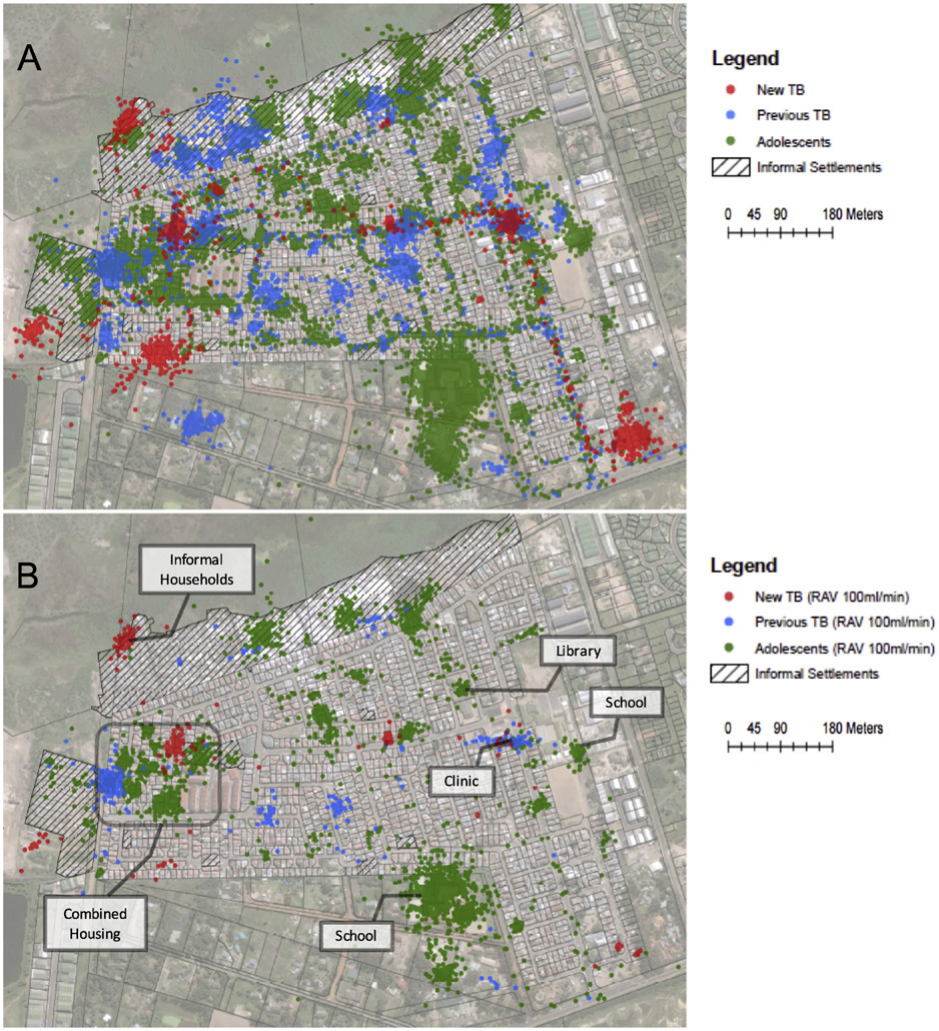
A. All GPS locations recorded by participants form the three groups overlaid on a map of community. B. GPS locations with rebreathed air volumes of 100 ml/min or greater.

Fig. 3A is a satellite image of Masiphumelele community. The GPS coordinates recorded for all participants throughout the period that they were monitored was added as a layer to this map. Each location data point was then linked to a calculated RAV per minute at the same time point and Fig. 3B shows only the data points linked to RAV per minute greater than or equal to 100 ml. Adding a further layer of physical structures allowed these clusters to be matched to their corresponding buildings and identified by those familiar with the community. This revealed several locations corresponding to public buildings including both of the community schools, the clinic and the library. Numerous households also remained both of the informal and brick-built type.

**Fig. 4:**
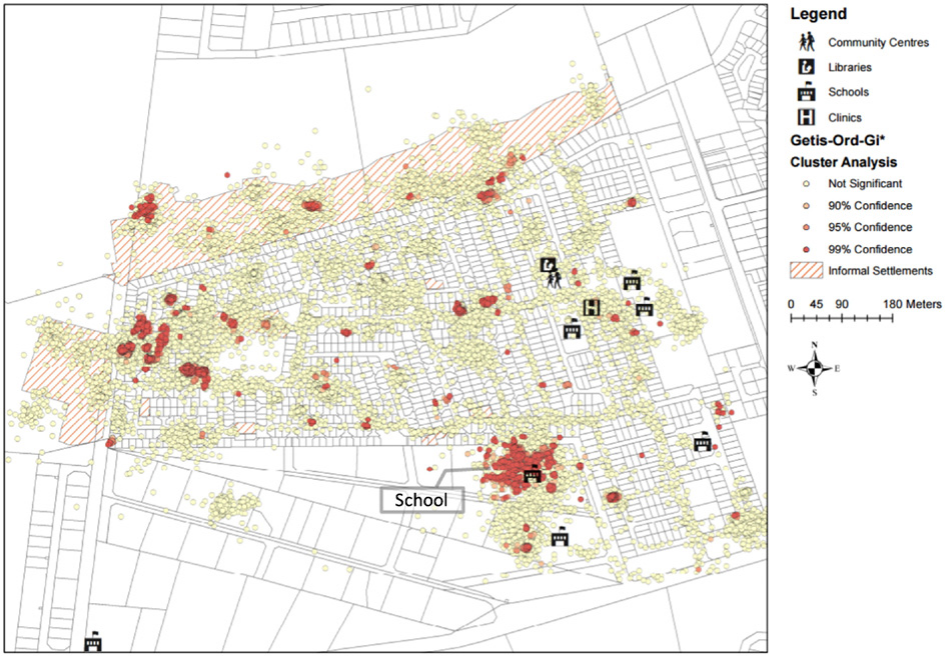
Getis-Ord-Gi* cluster analysis highlighting the school as a hot spot.

The combined GIS data points for all participants and all time points where then subjected to a hot spot analysis using an embedded analytical tool in ArcGIS. This generated an image of clusters of high RAV per minute distinct from surrounding space. From the public buildings with high RAV per minute identified in Fig. 3B only one of the schools was found to be a hot spot with a high degree of confidence (Fig. 4).

## 4 Discussion

In an indoor environment the risk to a susceptible individual of acquiring a new TB infection in the presence of an infectious source is proportional to the fraction of contaminated exhaled air and the duration of the exposure (Riley et al., 1978). A systematic approach to identifying transmission sites must be based on both these factors. By equipping our study participants with unobtrusive personal CO_2_/GIS monitoring devices we were able to integrate data regarding both the level of risk at different sites visited and the length of time an individual remained exposed to that risk. Applying a Getis-Ord-GI* cluster analysis (Getis and Ord, 1992) primarily located the community school as a potential TB hot spot.

Schools have long been recognized as sites of TB transmission (Bates et al., 1965). Recently a study from the KwaZulu Natal province in South Africa used site-based CO_2_ measurement and calculated annual probabilities of acquiring infection using estimates of exposure durations (Taylor et al., 2016). Both the classroom and also the clinic were found to have the highest annual probabilities of acquiring infection for school attendees and clinic staff respectively.

Our finding of high transmission *potential* at a school fits with the high acquisition rate of infection by school children found in cross-sectional tuberculin skin test data (Wood et al., 2010, Middelkoop et al., 2011). The force of infection for adolescents in the same South African community peaks in the mid-teens with an annual risk of infection of 7.9% in the pool of non-infected individuals (Wood et al., 2010). In recent years investigation of social networks in South Africa has demonstrated an increase in social mixing with a peak in median daily contacts for school age adolescents (Wood et al., 2012). These independent variables are synergistic with school building ventilation, classroom occupancy and exposure times which are encapsulated in RAV measurements. Taken together these elements may help to explain the high TB prevalence in South African young adults.

The critical problem of locating transmission has been tackled with a variety of methodologies previously. Retrospective interviews of genotype clustered-cases and subsequent mapping of shared social networks has been employed in rural Uganda (Chamie et al., 2015). A study in Cape Town used interviews and transect walks to identify public gathering places prospectively which were then ranked by a transmission risk grading system (Murray et al., 2009). Specific at-risk groups have also been investigated to determine the significance of their occupational exposures such as public transport workers in Lima, Peru (Horna-Campos et al., 2010) and healthcare workers in South Africa (Menzies and Joshi, 2007). These approaches have highlighted drinking venues, churches, marketplaces, buses, minibus taxis and clinics (Murray et al., 2009). Our study did not find these sites to be important. As a proportion of the total grouping together shops, drinking venues and churches only accounted for only minimal volumes of rebreathed air. This was predominantly due to short exposures at these locations when compared with much more prolonged exposures at other sites. This may partly be explained by an under-representation of healthy, employed adults in our sampled population and without a focus on at-risk workers.

The social behaviour measurements from our three groups demonstrated some distinct differences. Diary entries showed that adolescents were more likely to share the room in which they sleep and lived in homes with greater numbers of co-occupants. Additionally they recorded fewer hours sleeping and were more mobile throughout the day, experiencing far greater exposure to congregate settings. Consequently they were exposed to volumes of rebreathed air outside households more than five times higher than either of the two groups with clinical TB disease. Most strikingly we found that for the adolescents more than half of the daily RAV came from time spent at school.

This transmission potential derived from RAVs can be interpreted as both exposure potential for the susceptible and transmission potential for the infectious. In this regard adolescents are both highly at risk of exposure in the school setting but also highly likely to transmit should they become infectious. We found that those with newly diagnosed TB are behaviourally far more limited than the younger healthy cohort. TB infectiousness is known to rapidly diminish during effective treatment. It is therefore conceivable that a high rate of new infection amongst adolescents may create a pool of undiagnosed pauci-bacillary disease in the community. Given frequent prolonged exposures to the high risk school environment significant transmission may occur from sub-clinically infected individuals with even a low rate of infectious particle generation. We predict that efforts to minimise transmission in schools would be highly efficacious and interventional research should be conducted urgently.

### 4.1 Limitations

Several possible limitations should be taken into account when interpreting the results of this study. Firstly the outcome is very sensitive to the population studied. Potential at-risk groups such as clinic employees, shop owners or mini-bus taxi drivers were not investigated and so locations specific to these groups were not identified. Secondly external sources of CO_2_ are difficult to fully exclude and may confound the results. Our data showed no evidence that open flame use contributed significantly to the measured signal. However, CO_2_ from decaying organic material or exhaust fumes from vehicles or generators could contribute to localised increases in the CO_2_ level and are difficult to assess. We suspect these contributions would probably be small relative to the measured signal, nevertheless high outlier values should be interpreted cautiously with this methodology. Thirdly the use of CO_2_ as a surrogate for airborne infectious particle spread may be imperfect since droplet nuclei may not be as readily diffusible as has been discussed elsewhere (Wood et al., 2014). Finally a more finely tuned assessment of risk would incorporate estimates of TB prevalences for sub-populations at the locations identified based on demography.

## 5 Conclusion

Dynamic, portable CO_2_ and GIS monitoring by members of a high burden community provides a new methodology to locate sites with high potential risk of TB transmission. Application of this approach in a South African community highlighted several communal buildings and especially a local school. This sampling method could be used to investigate a range of communities which could differ in their sites of transmission risk. It may also be useful for investigating sub-groups within those communities. By avoiding the need to pre-select sites for investigation previously unrecognized hot spots may be discovered. Targeting sites revealed by this technique may improve the cost-effectiveness of structural or air-cleaning interventions.

## Figures and Tables

**Table 1:** Characteristics of participants of the three sub-groups Median and Interquartile ranges given unless otherwise stated. Categorical variables were compared using the Fisher exact test and continuous variables using a *t*-test when parametric and a Wilcoxon rank sum test (W) when nonparametric.

	New TB	Previous TB	Adolescents	*p*	
n	12	24	58		
Age (mean (s.d.))	33.42 (9.51)	34.42 (8.51)	16.36 (2.44)	< 0.001	
Sex (male %)	8 (66.7)	6 (25.0)	20 (34.5)	0.045	
HIV status (%)				< 0.001	
Negative	5 (41.7)	3 (12.5)	0 (0.0)		
Positive	7 (58.3)	17 (70.8)	0 (0.0)		
Unknown	0 (0.0)	4 (16.7)	58 (100.0)		
Employment or school (%)				< 0.001	
Full time	2 (16.7)	9 (37.5)	0 (0.0)		
Part time	4 (33.3)	4 (16.7)	0 (0.0)		
School	1 (8.3)	2 (8.3)	56 (96.6)		
Unemployed	5 (41.7)	9 (37.5)	2 (3.4)		
Housing type (informal %)	9 (75.0)	15 (62.5)	45 (77.6)	0.368	
Number of cohabitees (mean)	1.50 (1.88)	2.75 (2.82)	3.66 (1.80)	0.004	
Number sharing room to sleep (mean)	0.42 (0.67)	1.17 (1.05)	1.38 (1.17)	0.024	
Movements per day	8.75 [6.38, 10.12]	5.33 [4.14, 6.36]	12.83 [9.67, 17.25]	< 0.001	(W)
Sleep (hours per day)	11.96 [10.84, 13.40]	9.87 [8.90, 10.90]	9.50 [8.79, 10.49]	0.001	(W)
RAV — total	44.17 [15.15, 80.57]	39.75 [23.38, 55.44]	126.69 [91.02, 168.43]	< 0.001	(W)
RAV — during sleep	9.53 [0.00, 42.99]	17.20 [0.00, 22.05]	14.67 [3.34, 35.55]	0.625	(W)
RAV — in households	12.38 [10.92, 26.41]	7.72 [4.01, 13.34]	13.69 [9.09, 29.65]	0.044	(W)
RAV — work or school	0.00 [0.00, 0.00]	3.14 [0.00, 13.15]	71.26 [41.05, 103.01]	< 0.001	(W)
RAV — on transport	0.10 [0.00, 0.19]	0.00 [0.00, 2.24]	0.00 [0.00, 0.00]	0.004	(W)
RAV — other	1.52 [1.18, 1.80]	2.89 [1.84, 6.24]	2.94 [1.76, 5.69]	0.208	(W)

**Table 2:** Percentage of locations with recorded use of flame for either heating or cooking by the three groups.

Flame Use (%)	New TB	Previous TB	Adolescent	Total
*Household locations*				
Heating	25	8.3	20.7	17.5
Cooking	16.7	20.8	22.4	22.0

*Congregate locations*				
Heating	8.3	0.0	8.6	6.4
Cooking	8.3	8.3	12.1	10.6
